# The Acute Effect of Different Cluster Set Intra-Set Rest Interval Configurations on Mechanical Power Measures During a Flywheel Resistance Training Session

**DOI:** 10.3390/sports12120324

**Published:** 2024-11-27

**Authors:** Shane Ryan, Declan Browne, Rodrigo Ramirez-Campillo, Jeremy Moody, Paul J. Byrne

**Affiliations:** 1 Department of Health and Sport Sciences, South East Technological University, Kilkenny Road Campus, R93 V960 Carlow, Ireland; declan.browne@setu.ie (D.B.); paul.byrne@setu.ie (P.J.B.); 2 Exercise and Rehabilitation Sciences Laboratory, Faculty of Rehabilitation Sciences, School of Physical Therapy, Universidad Andres Bello, Santiago 7590000, Chile; rodrigo.ramirez@unab.cl; 3 Cardiff School of Sport and Health Sciences, Cardiff Metropolitan University, Cardiff CF5 2YB, UK; jmoody@cardiffmet.ac.uk; 4 School of Physical Education and Sports, Nisantasi University, Istanbul 34398, Turkey

**Keywords:** intra-set rest, fatigue, resistance training, power output, muscle strength, human physical conditioning

## Abstract

The aim of this study was to compare the acute effect of three cluster set (CS) intra-set rest intervals (15 s, 30 s, and 45 s) on mechanical performance measures during a flywheel resistance training session. Twelve amateur male field sport athletes attended three training measurement sessions (separated by 14 days of wash-out), consisting of four sets of nine repetitions (as cluster-blocks: 3 + 3 + 3), using a 0.050 kg·m^−2^ inertial load. The flywheel quarter-squat (QS) and the flywheel Romanian deadlift (RDL) were selected as resistance training exercises. Participants were randomly allocated different CS intra-set rest durations: 15 s, 30 s, or 45 s. The mean power (MP), peak concentric power (PP_con_), peak eccentric power (PP_ecc_), and eccentric overload (EO) were measured. A two-way (within–within) repeated-measures ANOVA reported that MP, PP_con_, PP_ecc_, and EO achieved similar values during the QS and RDL sessions involving 30 s and 45 s CS intra-set rest durations. It was noted that the first set did not always result in the greatest performance output for the 30 s and 45 s intervals. Compared to 15 s, the 30 s and 45 s CS intra-set rest durations showed greater MP, PP_con_, and PP_ecc_ during set 2 (all *p* ≤ 0.05), set 3 (all *p* < 0.001), and set 4 (all *p* < 0.001) for both QS and RDL, and greater EO in the QS exercise (the four sets combined). Compared to shorter (15 s) cluster set intra-set rest intervals, longer (30–45 s) configurations allow greater physical performance outcome measures during flywheel QS and RDL resistance training sessions. The implications for longer-term interventions merit further research.

## 1. Introduction

Resistance training (RT) is a fundamental component of field sport athletic preparation [1]. Indeed, muscle power production may determine playing level (elite and sub-elite status) and success rates among field sport athletes [1,2,3,4]. Similarly, athletes with greater maximal strength showed superior power production compared to their weaker counterparts [5,6]. Furthermore, the transfer of training stimuli to athletic performance may be the result of components of RT prescription (e.g., training load or type of muscle action) [7]. For example, compared to concentric (CON) and isometric (ISO) training, eccentric (ECC) training allows 20–50% greater loads and may lead to greater strength and power development [8,9].

Several architectural and neurological adaptations can be induced with ECC training [7,10], including (but not limited to) additional sarcomeres within a series, increased type II muscle fibre composition (%), hypertrophic effects (particularly in fast twitch muscle fibres), and muscle–tendon unit stiffness [10]. These adaptions can increase an athlete’s strength level and explosive capacity (i.e., rate of force development) more effectively compared to traditional resistance training (TRT) [8,9]. Furthermore, when implemented at high velocities, ECC training can increase elastic energy build-up within muscle tissues, subsequently enhancing the stretch-shortening cycle response and CON power output during sporting movements [10,11,12]. Maintaining maximal or near-maximal mechanical outputs during muscular actions (ECC and CON) of rapid velocity may elicit greater neuromuscular adaptative responses [7,10,13].

These neuromuscular responses may be accomplished with flywheel resistance training (FRT), which has been shown to be effective in improving strength [8], power [14], and agility properties in amateur and professional athletes [15]. The ability of FRT to utilize rotational momentum through a weighted disc attached to the user via a harness and tether allows for the generation and transfer of kinetic energy during ECC exercise [16,17]. Moreover, compared to traditional barbell resistance training, FRT allows higher CON velocities and ECC muscular activity [17,18], peak mechanical power, and eccentric overload (EO) stimulus [13,19,20]. However, FRT can induce increased levels of fatigue [21,22].

A cluster set (CS) training approach allows set manipulation [23], incorporating brief ‘intra-set’ rest intervals to reduce the onset of fatigue, increasing barbell velocity, movement efficiency, and mechanical power outputs, while reducing the rating of perceived exertion during TRT [24,25]. However, CS effectivity during FRT is under-researched. In a series of FRT cross-sectional experiments, Ryan et al. [26,27] confirmed that CSs (using 45 s intra-set rest periods) allowed maximal mechanical performance (i.e., no drop-off in performance was noted across sets). However, due to the insufficient FRT literature assessing the implications of increased fatigue associated with ECC muscular contractions (in comparison to CON and ISO contractions) [28], the maximal CS configuration during FRT is yet to be identified. A systematic review and meta-analysis by Davies et al. [29] assessed alternative traditional cluster set (TCS) rest configurations, reporting that intra-set rest intervals of 30 s and 45 s allowed better strength and power responses, whereas durations of 15 s may be preferred for strength-endurance training [29].

Although CS configurations during TRT have been reported to lead to several training response differences dependent on the intra-set rest duration [24,29], the extrapolation of such findings to FRT can be inappropriate. Therefore, the aim of this study was to compare the acute effect of three CS intra-set rest intervals (15 s, 30 s, and 45 s) on the performance measures of mean power (MP), peak CON power (PP_con_), peak ECC power (PP_ecc_), and EO during an FRT session. The hypothesis of this study was that the duration of the intra-set rest interval would play a significant role in an athletes’ ability to maintain FRT performance. Specifically, we hypothesize that longer intra-set rest intervals would allow greater maintenance of performance during FRT.

## 2. Materials and Methods

### 2.1. Study Design

**Figure 1:**
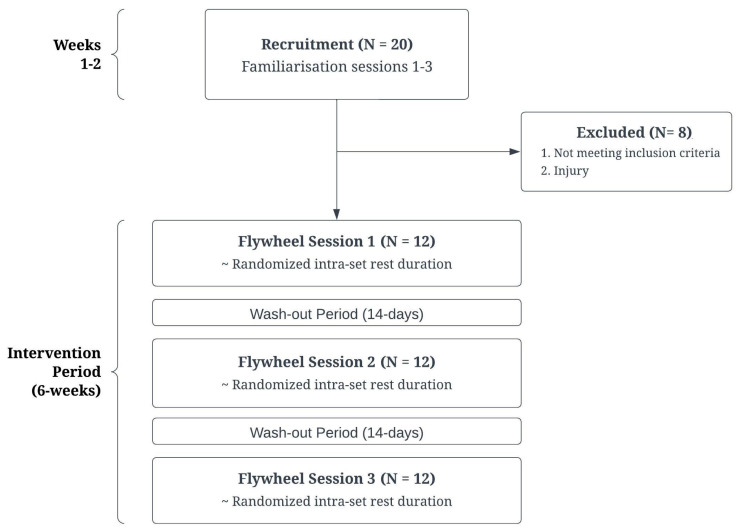
Study design for intra-set rest duration intervention.

This study applied a randomized cross-over design, where participants attended 3 flywheel training sessions, each separated by 14 days. Participants were male field sport athletes, who were assessed to determine differences in performance measures related to flywheel cluster set training using various intra-set configurations; 15 s, 30 s, and 45 s (Figure 1).

### 2.2. Participants

Twenty amateur male collegiate field sport athletes (age, 24.3 ± 4.2 years; weight, 83.6 ± 9.4 kg; height, 1.79 ± 0.07 m) volunteered to participate in this study. However, twelve participants completed the study, due to eight participants been excluded due to not meeting the inclusion criteria or becoming injured separately to the training intervention. Participants were required to have ≥2 years of resistance training experience with no previous flywheel training experience. Testing was conducted during the off-season phase of participants’ training calendar, to not disrupt participants’ training. At the time of testing, all participants reported no previous orthopedic or musculoskeletal injuries to the lower extremities within the previous 6 months based upon medical screening. Additionally, during this time, participants were given a briefing of the testing procedures, associated benefits, and possible risks prior to giving informed consent to participate. An a priori GPower sample size calculation was conducted for a two-way repeated-measures ANOVA, with an effect size = 0.60 [30], an α error probability of <0.05, the non-sphericity correction € = 1, the correlation between the repeated measures = 0.5, and a desired power (1-ß error) = 0.95. The sample size calculation resulted in a total sample size of 12 participants. Experimental proceedings were conducted according to the Declaration of Helsinki, and the experimental protocol was approved by the Research Ethics Committee of the ***blind for peer review*** University, ***blind for peer review*** (application number 316).

### 2.3. Intervention

Upon study recruitment, participants underwent three FRT familiarization sessions as per recommendations by Ryan et al. [26,27]. Thereafter, upon arrival for each FRT session, participants were randomly allocated to 1 of 3 intra-set rest configurations: 15 s, 30 s or 45 s. The flywheel cluster training protocol consisted of QS and RDL exercises (always in the same order) for 4 sets of 9 repetitions in clusters of 3 (3 × 3 × 3). Inter-set rest lasting 4 min was provided between each set for both exercises. Participants attended FRT sessions fourteen days apart as a wash-out period to prevent participants from adapting to the FRT stimulus. Thereafter, each participant followed the same testing protocol using a different intra-set rest duration. Thus, the intervention period lasted 6 weeks (including FRT sessions and wash-out periods). Prior to each FRT session, participants underwent a warm-up protocol consisting of 5 min self-paced jogging, followed by 5 dynamic stretches (quadriceps, hamstrings, gluteal, adductors, and gastrocnemius) performed over a 10 m distance consisting of 14 repetitions per leg [31].

### 2.4. Mechanical Range of Motion

Range of motion (ROM) was standardized for participants during the QS and RDL exercises using a goniometer (Large goniometer, Cartwright fitness, UK) and tape to ensure consistent depth and ROM. During the QS exercise, participants began the exercise in full knee extension position (180°), then moved to a depth position resulting in a knee joint angle of 135°. Similarly, during the RDL exercise, participants were instructed to begin the movement from a full knee extension position (180°), lowering forward with a “neutral spine and slight bend in their knees” to a depth where they could touch the bar on the tape placed as a marker limiting the frontal depth to the lower aspect of the patella. Moreover, participants were instructed to perform all repetitions with maximal CON force output and delay the breaking action of ECC forces until the final third of the ECC phase, in an attempt to maximize EO [32]. An inertial load of 0.05 kg·m^−2^ was used for all repetitions during both QS and RDL exercises. Ankle plantar flexion was prohibited during exercise.

### 2.5. The kMeter Application

The kMeter application (app) has been reported as a viable means of tracking and recording flywheel training parameters [33]. Therefore, the app was employed to assess CON/ ECC training parameters during each intra-set rest configuration. Additionally, the mean and peak power scores were recorded to assess drop-offs in FRT performance, which could alter the desired training effect. Furthermore, EO was calculated in both absolute (Nm = peak CON/ ECC force) and relative values (ECC peak force/100/CON peak force/100) [33].

### 2.6. Statistical Analysis

Using a Shapiro–Wilk test, the normality of variables was verified. A two-way (within–within) repeated-measures (sets 1, 2, 3, and 4) ANOVA was used to compare between groups (CS intra-set intervals: 15 s, 30 s, 45 s) in both QS and RDL exercises. Additionally, a within-group analysis was used to assess differences in training measures for each individual intra-set group across the four timepoints (set 1–4). Pairwise post hoc analysis using a Bonferroni adjustment were carried out to investigate between group differences. The interclass correlation coefficient (ICC) was reported for each intra-set rest interval to analyze the peak and mean power output across sets. The interpretation of ICC values was poor (0.00–0.49), moderate (0.50–0.69), high (0.70–0.89), or very high (≥0.9) [34]. Absolute reliability was assessed using the coefficient of variance (CV%), calculated as standard deviation/mean × 100 [35]. Cohen’s d effect size (ES) was calculated and interpreted as trivial (<0.2), small (0.2–0.5), moderate (>0.5–0.8), or large (>0.8) [36]. The ES was used to estimate the difference between performance measures (MP, PP_con_, PP_ecc_, and EO) across sets (set 1–4) for each intra-set rest interval group. Statistical significance was established at *p* ≤ 0.05. Statistical analyses were carried out using the software package IBM SPSS (Version 27, SPSS Inc., Chicago, IL, USA).

## 3. Results

The descriptive data (mean, standard deviation) of training measures (MP, PP_con_, PP_ecc_, and EO) are displayed in Table 1.

**Figure 2:**
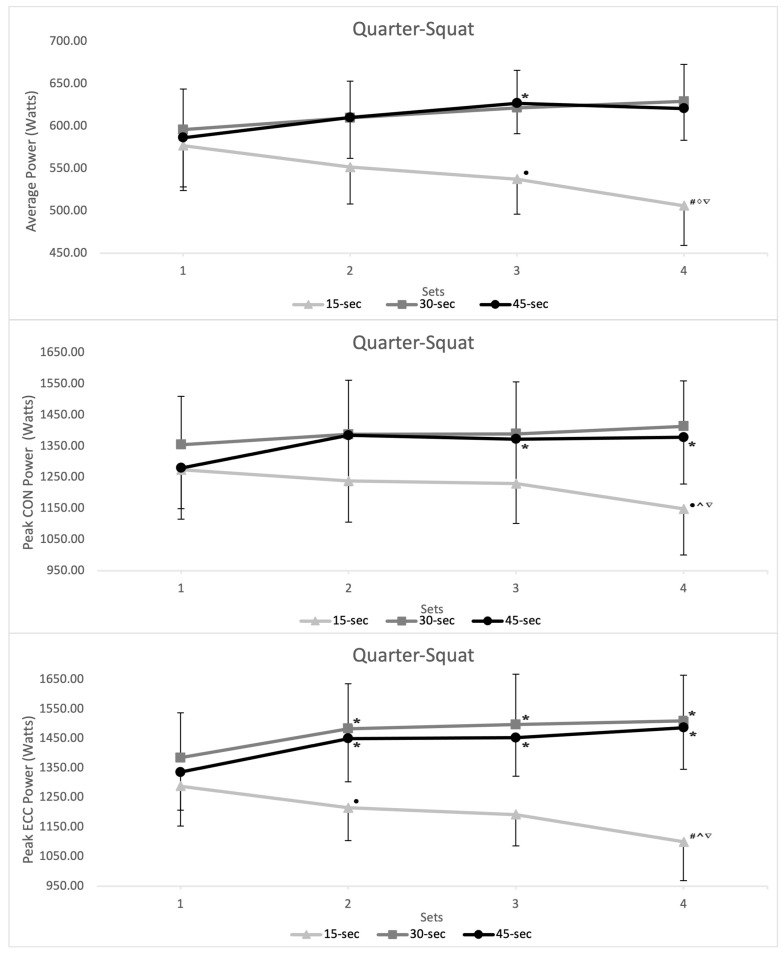
Inter-day inter-set mean power (MP), peak concentric power (PP_con_), and peak eccentric (PP_ecc_) output (watts) by intra-set rest duration during the flywheel quarter-squat exercise from sessions 1 to 4. * = significantly greater than day 1 (*p* ≤ 0.05); ● = significantly less than day 1 (*p* ≤ 0.05); # = significantly less than day 1 (*p* ≤ 0.001); ^ = significantly less than the result from set 2 (*p* ≤ 0.05); ◊ = significantly less than the result from set 2 (*p* ≤ 0.001); ∇ = significantly less than the result from set 3 (*p* ≤ 0.05).

**Figure 3:**
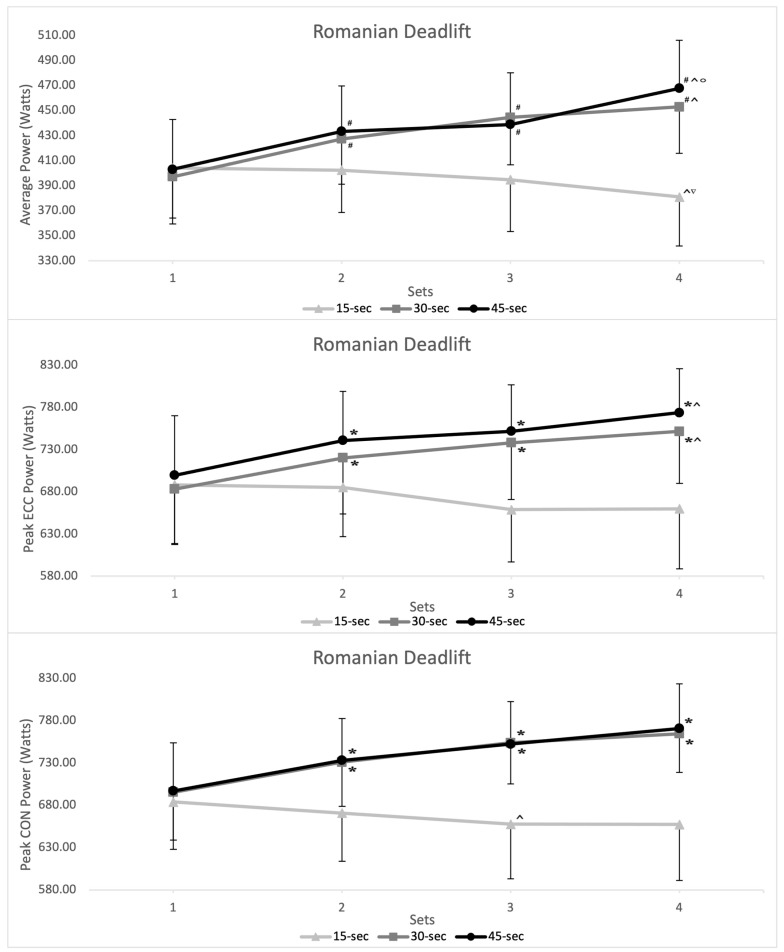
Inter-day inter-set mean power (MP), peak concentric power (PP_con_), and peak eccentric (PP_ecc_) output (watts) by intra-set rest duration during the flywheel quarter-squat exercise from sessions 1 to 4. * = significantly greater than day 1 (*p* ≤ 0.05); # = significantly greater than day 1 (*p* ≤ 0.001); ^ = significantly less than the result from set 2 (*p* ≤ 0.05); ° = significantly greater than the result from set 3 (*p* ≤ 0.001).∇ = significantly less than the result from set 3 (*p* ≤ 0.05).

Intra-set interval duration analysis reported no significant differences in performance measures (MP, PP_con_, and PP_ecc_) between intra-set intervals for set 1. However, compared to the 15 s intra-set rest interval, the 30 s and 45 s intra-set rest intervals showed greater MP, PP_con_, and PP_ecc_ for set 2, 3, and 4, for both QS and RDL exercises (Figure 2; Figure 3).

A paired-samples *t*-test revealed no differences in performance measures comparing the 30 s and 45 s intra-set rest intervals for both the QS (*p* = 0.468–0.991) and RDL (*p* = 0.189–0.921) exercises across MP, PP_con_, and PP_ecc_ measures. With comparable ICC and CV% reported between sets 1 and 4 for both QS (30 s—ICC = 0.976–0.994, CV% = 7.76–11.79; 45 s—ICC = 0.991–0.994, CV% = 7.67–11.73) and RDL (30 s—ICC = 0.985–0.994, CV% = 7.01–8.94; 45 s—ICC = 0.989–0.994, CV% = 7.35–9.16) exercises. However, multiple large–very-large ESs were reported in the later sets, when comparing the 30 s and 15 s intra-set rest interval and 45 s and 15 s intra-set rest interval for both the QS and RDL exercises across the MP, PP_con_, and PP_ecc_ measures (see Table 2). Additionally, post hoc analysis reported multiple differences (*p* = 0.05 and *p* < 0.001) in performance measures between sets for the 15 s intra-set rest interval (see Figure 2; Figure 3).

For the QS exercise, the 15 s intra-set rest interval showed lower MP during set 2 (vs. 30 s, *p* = 0.009; vs. 45 s, *p* = 0.007), set 3 (vs. 30 s, *p* < 0.001; vs. 45 s, *p* < 0.001) and set 4 (vs. 30 s, *p* < 0.009; vs. 45 s, *p* < 0.001). Similar differences were reported in PP_con_ during set 2 (vs. 30 s, *p* = 0.007; vs. 45 s, *p* = 0.008), set 3 (vs. 45 s, *p* = 0.010) and set 4 (vs. 30 s, *p* = 0.028; vs. 45 s, *p* < 0.001) and PP_ecc_ during set 2 (vs. 30 s, *p* = 0.003; vs. 45 s, *p* = 0.003), set 3 (vs. 30 s, *p* < 0.001; vs. 45 s, *p* < 0.001) and set 4 (vs. 30 s, *p* < 0.001; vs. 45 s, *p* < 0.001). The EO also was lower for the 15 s intra-set rest interval during set 3 (vs. 30 s, *p* = 0.045; vs. 45 s, *p* = 0.015) and during set 4 (vs. 30 s, *p* = 0.043; vs. 45 s, *p* = 0.001).

**Figure 4:**
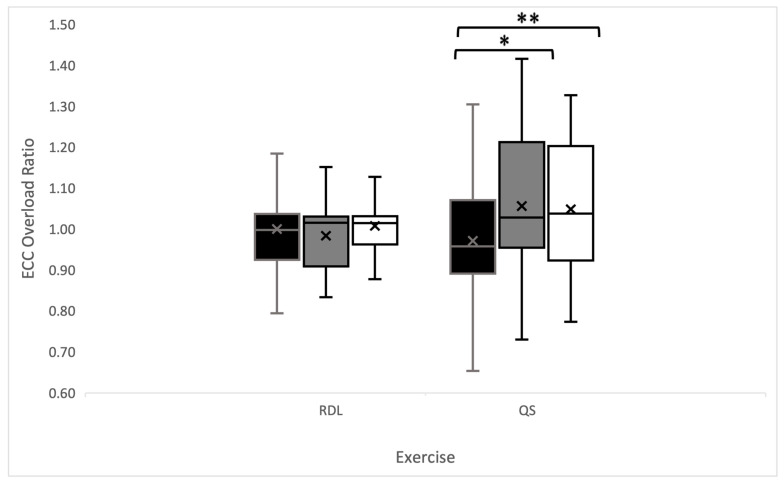
A comparison of the intra-set rest intervals (15 s (black), 30 s (grey), and 45 s (white)) by the eccentric overload generated from peak concentric and eccentric outputs (watts) for the Romanian deadlift and quarter-squat exercise (* *p* = 0.05; ** *p* < 0.001).

For the RDL exercise, the 15 s intra-set rest interval showed significant differences in MP during set 2 (vs. 30 s, *p* = 0.012; vs. 45 s, *p* = 0.005), set 3 (vs. 30 s, *p* < 0.001; vs. 45 s, *p* < 0.001) and set 4 (vs. 30 s, *p* < 0.001; vs. 45 s, *p* < 0.001). Similar differences were reported in PP_con_ output during set 2 (vs. 30 s, *p* = 0.004; vs. 45 s, *p* < 0.001), set 3 (vs. 30 s, *p* < 0.001; vs. 45 s, *p* < 0.001), and set 4 (vs. 30 s, *p* < 0.001; vs. 45 s, *p* < 0.001). And PP_ecc_ output during set 2 (vs. 45 s, *p* = 0.037), set 3 (vs. 30 s, *p* < 0.001; vs. 45 s, *p* < 0.001), and set 4 (vs. 30 s, *p* < 0.001; vs. 45 s, *p* < 0.001). Trivial–small differences were noted in EO during the RDL exercise across intra-set rest groups (see Figure 4).

## 4. Discussion

The main findings of this study indicate that intra-set rest intervals play a significant role in a user’s ability to maintain mechanical power output during an FRT session, with consistent findings present among both QS and RDL exercises. Additionally, if the intra-set rest interval is insufficient in duration, decrements in mechanical power output (MP, PP_con_, and PP_ecc_) will arise as the session persists. Further, the EO during FRT seems dependent on the intra-set rest duration during the QS exercise.

Maximizing the transfer of resistance training to athletic performance requires an understanding of movement patterns, loads, and velocities during training sessions [1]. To date, the FRT literature has investigated inertial loading parameters, improving the understanding of CON and ECC variables [13,37]. However, the literature examining the effect of fatigue during FRT and the influence of fatigue accumulation on training measures related to strength and power development remains scarce. The results of this study indicate that the introduction of brief intra-set rest periods (30 s and 45 s protocols) proved beneficial in minimizing the magnitude of power reductions associated with FRT. However, if the intra-set rest interval is insufficient in duration, drop-offs in MP and peak mechanical (PP_con_ and PP_ecc_) outputs will ensue. Indeed, the intra-set rest frequency and duration modifications may affect training outcomes, with intra-set rest durations of 30 s and 45 s enhancing strength and power performance, while shorter intra-set rest durations may be more beneficial for strength-endurance phases of training [29]. The 15 s intra-set rest protocol showed significant decreases in MP during set 3 and set 4 for the QS exercise and set 4 for the RDL exercise. The reduction in MP resulted in significant decrements in CON and ECC power capacity during set 4 for the QS exercise and ECC power capacity during set 3 of the RDL exercise. Moreover, when comparing intra-set rest interval protocols, significant differences were found in the ability to attain an EO contraction (15 s vs. 30 s and 15 s vs. 45 s) during the QS exercise in set 2, set 3, and set 4. Additionally, no drop-offs in performance measures (MP, PP_con_, PP_ecc_, and EO) were observed using the 30 s and 45 s intra-set rest protocols across all sets.

Presently, there is a lack of FRT literature incorporating cluster sets with which we can compare the results from the current study. However, numerous TRT studies have investigated the benefits of CS structures, which may transfer over to the flywheel model. A systematic review by Tufano et al. [25] highlighted the importance of intra-set rest duration on the ability of athletes to attenuate force and velocity loss during TRT protocols. Additionally, Tufano et al. [25] reported that shorter intra-set rest durations negatively impact an athlete’s ability to prevent performance loss during exercise, especially during the later portions of sets. This is especially important due to the nature of the 15 s protocol in the current study failing to attain an EO stimulus in the later sets (set 3 and set 4) of the QS exercise. Furthermore, a recent systematic review reported intra-set rest durations between 30 and 45 s to be the most beneficial during the strength and power phases of RT programmes, whereas a shorter intra-set rest duration (i.e., 15 s) may be more beneficial during the strength-endurance phases of training, where training volume and work capacity represent the goal [29].

To date, FRT has been proven to enhance a variety of athletic components, including speed, strength, and power, largely due to the ability to elicit an EO contraction [8,15]. A topical review by Tesch et al. [25] summarized the set configurations of several FRT intervention studies, concluding that four sets of seven repetitions using the 0.050 kg·m^−2^ inertial load was the most used scheme. However, many of the studies included within the meta-analysis were conducted on single-joint movements or small muscle groups, whereas large multi-joint movements are frequently preferred for strength and power development [38,39]. Similarly, a systematic review by Allen et al. [40] reported set configuration findings from eleven FRT studies, suggesting 1–2 sessions per week consisting of 5–10 repetitions can increase athletic performance in male soccer players. However, conflicting reports by Sabido et al. [22] highlighted significant decrements in mechanical power (peak CON/ECC) outputs during a quarter-squat (multi-joint) exercise independent of the inertial load (0.025 kg·m^−2^, 0.050 kg·m^−2^, 0.075 kg·m^−2^, or 0.100 kg·m^−2^) following 1 set of 10 repetition sequences (e.g., with repetition 5 using 0.050 kg·m^−2^ inertial load). This suggests that the onset of fatigue during continuous repetition sequences may disrupt the later portion of repetitions in the suggested models from Tesch et al. [25] and Allen et al. [40]. Additionally, although such set configurations have been proven to positively impact sporting performance, a limitation of the literature is that the vast majority of FRT studies fail to include training variable data (peak/mean CON and ECC outputs) within reports [41]. Thus, FRT measures (peak/mean power outputs) play a crucial role in an athlete’s ability to determine whether an EO contraction is achieved [13]. The application of CS within the flywheel paradigm could potentially negate the commonly reported neuromuscular fatigue induced drop-off in FRT performance [37].

The current study presents some limitations. First, the current study was conducted on male field sport athletes, so the transfer of results to other populations is yet to be understood. Future research should investigate the effects of intra-set rest duration on other populations (female, elderly, etc.). Additionally, this study assessed the difference between three intra-set rest durations on training measures, reporting significant decreases in MP, PP_con_, and PP_ecc_ using the 15 s intra-set rest interval. This may be due to the limited sample size with 12 participants taking part in this research study. Future research may consider investigating the comparison of a continuous straight set repetition scheme versus a cluster set training modality.

The findings reported in this study provide practical applications for strength and conditioning coaches when implementing flywheel cluster set protocols. The ability for users to maintain peak and mean power outputs does not differ between 30 s and 45 s intra-set rest durations, although the implementation of a 15 s intra-set rest interval proved detrimental for training performance, as training measures (MP, PP_con_, and PP_ecc_) showed significant decrements in performance the longer the training session persisted (i.e., in set 3 and set 4).

## 5. Conclusions

In conclusion, it is recommended that practitioners utilize 30 s and 45 s intra-set rest durations when implementing flywheel cluster training with athletes, if PP_con_, PP_ecc_, and EO are the desired training effects. If the intra-set rest period is not sufficient in duration, then the ability of the CS to mitigate fatigue may be diminished, resulting in a reduction in performance characteristics (MP, PP_con_, PP_ecc_, and EO) related to FRT.

## Figures and Tables

**Table 1:** Inter-set changes in training measures.

**Quarter-Squat**				
**Mean Power (Watts)**	Set 1	Set 2	Set 3	Set 4
15 s	577.00 ± 53.04	551.67 ± 43.68	537.58 ± 41.39	506.00 ± 46.66
30 s	595.74 ± 47.90	609.81 ± 43.02	621.47 ± 44.38	629.06 ± 43.65
45 s	586.19 ± 58.00	610.11 ± 47.99	626.92 ± 35.76	620.84 ± 37.49
**Peak CON Power (Watts)**				
15 s	1273.58 ± 124.94	1237.67 ± 132.12	1229.75 ± 127.61	1148.33 ± 147.42
30 s	1354.66 ± 155.53	1387.83 ± 173.72	1389.05 ± 166.94	1413.27 ± 145.73
45 s	1279.75 ± 164.22	1384.32 ± 161.32	1372.30 ± 141.10	1378.35 ± 149.67
**Peak ECC Power (Watts)**				
15 s	1288.17 ± 135.31	1214.58 ± 110.36	1191.75 ± 105.97	1099.67 ± 130.90
30 s	1384.69 ± 153.10	1483.24 ± 152.05	1497.21 ± 170.83	1509.45 ± 155.10
45 s	1335.41 ± 128.54	1449.98 ± 147.12	1452.77 ± 131.44	1487.09 ± 141.69
**CON/ECC Ratio (Watts)**				
15 s	1.00 ± 0.14	0.97 ± 0.13	0.95 ± 0.16	0.95 ± 0.17
30 s	1.01 ± 0.17	1.07 ± 0.19	1.08 ± 0.17	1.07 ± 0.14
45 s	1.04 ± 0.18	1.04 ± 0.14	1.05 ± 0.14	1.07 ± 0.15
**Romanian Deadlift**				
**Mean Power (Watts)**	Set 1	Set 2	Set 3	Set 4
15 s	403.92 ± 39.96	402.33 ± 33.79	394.75 ± 41.53	380.83 ± 39.13
30 s	397.04 ± 37.54	427.27 ± 36.15	444.47 ± 37.93	452.85 ± 37.09
45 s	402.98 ± 39.79	433.26 ± 36.44	438.84 ± 41.13	467.69 ± 38.41
**Peak CON Power (Watts)**				
15 s	683.69 ± 55.80	670.58 ± 56.70	657.42 ± 64.13	657.08 ± 65.76
30 s	695.10 ± 56.26	730.84 ± 52.22	753.79 ± 48.88	764.61 ± 46.11
45 s	696.81 ± 57.06	732.95 ± 49.71	752.31 ± 50.10	770.71 ± 52.93
**Peak ECC Power (Watts)**				
15 s	688.33 ± 70.64	685.08 ± 58.18	658.92 ± 62.13	660.00 ± 71.34
30 s	683.29 ± 64.71	720.16 ± 66.31	738.07 ± 67.25	751.55 ± 61.49
45 s	699.64 ± 70.35	740.85 ± 58.03	751.74 ± 55.19	773.70 ± 52.30
**CON/ECC Ratio (Watts)**				
15 s	1.00 ± 0.11	1.01 ± 0.12	0.99 ± 0.11	0.99 ± 0.13
30 s	0.98 ± 0.08	0.99 ± 0.08	0.98 ± 0.09	0.99 ± 0.09
45 s	1.01 ± 0.11	1.01 ± 0.09	1.00 ± 0.09	1.01 ± 0.11

Mean power output, peak concentric power, peak eccentric power, and CON/ ECC ratio for eccentric overload (W; mean ± standard deviation) by intra-set rest duration and training set. Coefficient of variance (CV%) and 95% confidence intervals (CIs) for each intra-set rest duration’s combined 4-set power output.

**Table 2:** Effect sizes between the three intra-set rest intervals for the four sets of cluster sets.

**Quarter-Squat**				
**Mean Power (Watts)**	Set 1	Set 2	Set 3	Set 4
15 s vs. 30 s	0.37	1.34	1.96	2.72
15 s vs. 45 s	0.17	1.27	2.31	2.71
30 s vs. 45 s	−0.18	0.07	0.14	−0.20
**Peak CON Power (Watts)**				
15 s vs. 30 s	0.57	0.97	1.07	1.81
15 s vs. 45 s	0.04	0.99	1.06	1.55
30 s vs. 45 s	−0.47	−0.02	−0.11	−0.24
**Peak ECC Power (Watts)**				
15 s vs. 30 s	0.67	2.02	2.15	2.86
15 s vs. 45 s	0.36	1.81	2.19	2.84
30 s vs. 45 s	−0.35	−0.22	−0.29	−0.15
**CON/ ECC Ratio (Watts)**				
15 s vs. 30 s	0.06	0.61	0.79	0.77
15 s vs. 45 s	0.25	0.52	0.67	0.75
30 s vs. 45 s	0.17	−0.18	−0.19	0.00
**Romanian Deadlift**				
**Mean Power (Watts)**	Set 1	Set 2	Set 3	Set 4
15 s vs. 30 s	−0.18	0.71	1.25	1.89
15 s vs. 45 s	−0.02	0.88	1.07	2.24
30 s vs. 45 s	0.15	0.17	−0.14	0.39
**Peak CON Power (Watts)**				
15 s vs. 30 s	0.20	1.11	1.69	1.89
15 s vs. 45 s	0.23	1.17	1.65	2.24
30 s vs. 45 s	0.03	0.04	−0.30	0.12
**Peak ECC Power (Watts)**				
15 s vs. 30 s	−0.07	0.56	1.22	1.37
15 s vs. 45 s	0.18	0.96	1.58	1.82
30 s vs. 45 s	0.27	0.33	0.22	0.39
**CON/ECC Ratio (Watts)**				
15 s vs. 30 s	−0.21	−0.20	−0.10	0.00
15 s vs. 45 s	0.09	0.00	0.10	0.17
30 s vs. 45 s	0.31	0.23	0.22	0.20

Mean power output, peak concentric power, peak eccentric power and CON/ECC ratio for eccentric overload (W; mean ± standard deviation). Effect size (ES) by intra-set rest durations and training set.

## Data Availability

The data presented in this study are available upon request from the corresponding author.
